# Pharmacological Approaches to Hearing Loss

**DOI:** 10.1124/pharmrev.124.001195

**Published:** 2024-11

**Authors:** Christopher R. Cederroth, Jonas Dyhrfjeld-Johnsen, Barbara Canlon

**Affiliations:** Department of Physiology and Pharmacology, Karolinska Institute, Stockholm, Sweden (C.R.C., B.C.); Translational Hearing Research, Tübingen Hearing Research Center, Department of Otolaryngology, Head and Neck Surgery, University of Tübingen, Tübingen, Germany (C.R.C.); and Acousia Therapeutics GmbH, Tübingen, Germany (J.D.-J.)

## Abstract

Hearing disorders pose significant challenges to individuals experiencing them and their overall quality of life, emphasizing the critical need for advanced pharmacological approaches to address these conditions. Current treatment options often focus on amplification devices, cochlear implants, or other rehabilitative therapies, leaving a substantial gap regarding effective pharmacological interventions. Advancements in our understanding of the molecular and cellular mechanisms involved in hearing disorders induced by noise, aging, and ototoxicity have opened new avenues for drug development, some of which have led to numerous clinical trials, with promising results. The development of optimal drug delivery solutions in animals and humans can also enhance the targeted delivery of medications to the ear. Moreover, large genome studies contributing to a genetic understanding of hearing loss in humans combined with advanced molecular technologies in animal studies have shown a great potential to increase our understanding of the etiologies of hearing loss. The auditory system exhibits circadian rhythms and temporal variations in its physiology, its vulnerability to auditory insults, and its responsiveness to drug treatments. The cochlear clock rhythms are under the control of the glucocorticoid system, and preclinical evidence suggests that the risk/benefit profile of hearing disorder treatments using chronopharmacological approaches would be beneficial. If translatable to the bedside, such approaches may improve the outcome of clinical trials. Ongoing research into the molecular and genetic basis of auditory disorders, coupled with advancements in drug formulation and delivery as well as optimized timing of drug administration, holds great promise of more effective treatments.

**Significance Statement:**

Hearing disorders pose significant challenges to individuals and their overall quality of life, emphasizing the critical need for advanced pharmacological approaches to address these conditions. Ongoing research into the molecular and genetic basis of auditory disorders, coupled with advancements in drug delivery procedures and optimized timing of drug administration, holds the promise of more effective treatments.

## I. Introduction

A recent report published by the Global Burden of Disease study predicted that by 2050 an estimated 2.4 billion individuals worldwide will suffer from hearing loss (Global Burden of Disease Hearing Loss Collaborators, 2021). This is nearly twofold more than the numbers predicted for diabetes (Global Burden of Disease Diabetes Collaborators, 2023). Thus, it is not surprising that hearing loss ranks in the top three most common causes of years living with disability, after low back pain and migraine (Global Burden of Disease Hearing Loss Collaborators, 2021). Hearing loss in younger children impedes the development of spoken language and is a major risk factor for dementia in older adults (Thomson et al., 2017; Livingston et al., 2020). The emotional consequences of hearing loss include depression and anxiety, loneliness, and isolation. Hearing loss can range from mild to profound and has multiple origins (e.g., childhood illnesses, pregnancy-related illnesses, injury, genetics, age, ototoxicity, and excessive or prolonged exposure to noise). Hearing aids and cochlear implants have dominated the rehabilitation landscape for hearing loss, albeit by principally providing an amplification of sound intensity for hearing aids or a restored stimulation of the auditory nerve for cochlear implants, without completely addressing the complexity of temporal and spectral sound decoding. With regards to cochlear implants, only 5% of those who qualify receive the intervention (De Raeve, 2016; Raine et al., 2016). Despite these global unmet needs, hearing loss has received substantially less governmental funding to address knowledge gaps than diabetes has (Cederroth et al., 2013). This financing gap is more evident in Europe than in the United States. This is possibly due to the Americans with Disabilities Act, which includes hearing loss as a disability, whereas Europe lacks such broad and detailed definitions of disability and leaves it up to each country to determine their own legislation and its interpretation (Vanhala, 2015). The last decade has witnessed increasing efforts to develop the potential use of therapeutic interventions, other than hearing aids and cochlear implants, such as gene therapy, brainstem or cortical implants, and pharmacological treatments. In this review, we seek to provide a snapshot of the current knowledge on the pharmacology of hearing loss, both the causes and treatments, with the intent to establish a basis from which new opportunities may emerge.

## II. A Primer on Cochlear Anatomy, Structure, and Function

The cochlea is a spiral-shaped coiled structure located in the inner ear and is responsible for converting sound vibrations into electrical signals that can be processed by the brain. The cochlea is divided into three fluid-filled chambers: the scala vestibuli, the scala media, and the scala tympani. The scala vestibuli and the scala tympani contain perilymph, which has a composition similar to cerebrospinal fluid, with a high concentration of sodium (140 mM) and a low level of potassium (5 mM). The scala media contains endolymph, which is high in potassium (150 mM) and low in sodium (1 mM). The scala vestibuli and scala tympani are sealed to the oval window and the round window, respectively. The altered ability of the external ear canal or middle ear to transfer sound waves from the ear canal to the inner ear is called conductive hearing loss.

The process of hearing begins when sound waves enter the ear and travel through the ear canal. These sound waves cause the eardrum to vibrate, which, in turn, sets the ossicles (malleus, incus, and stapes) in the middle ear into motion. The movement of the ossicles transmits sound vibrations to the cochlea through the oval window. There are several dysfunctions that can cause conductive hearing loss such as otitis media (middle ear inflammation), otosclerosis (a heritable condition that leads to an extensive ossification of the middle ear bones), and tympanic membrane rupture (e.g., either by noise blasts or mechanical damage).

Inside the cochlea, the mechanical vibrations are converted into electrical signals by the hair cells. These signals are then transmitted to the brain along the cochlear nerve for processing, ultimately allowing us to perceive and interpret sound. There are two types of hair cells: a single row of inner hair cells (IHCs) and three rows of outer hair cells (OHCs). IHCs are sensory cells that transmit sound information to the spiral ganglion neurons (SGNs) and then to the brain. OHCs contain prestin, which is a voltage-sensitive membrane motor protein that is responsible for amplification of sound-induced vibrations within the cochlea (Zheng et al., 2000). Compared with the IHCs, the OHCs are more sensitive to noise trauma, ototoxic drugs, and age-induced hearing loss. Loss of OHCs results in reduced hearing sensitivity and frequency discrimination. The IHC overall appears to be more resistant to cell death than the OHC is. However, the presynaptic region of the IHCs is particularly sensitive to damage induced by noise overstimulation and aging. The basal pole of the IHC contains synaptic ribbons that are responsible for the release of the neurotransmitter glutamate and the activation of the auditory nerve (Glowatzki and Fuchs, 2002). Cochlear synaptopathy induced by noise trauma or aging results in a reduction of synaptic ribbons, causing acute and irreversible hearing loss (Kujawa and Liberman, 2009; Sergeyenko et al., 2013) without any morphological alteration to the IHCs. Cochlear synaptopathy is thought to lead to difficulties in understanding speech in noisy environments, without showing alterations in hearing thresholds (Bakay et al., 2018; Monaghan et al., 2020).

### A. The Mechanotransduction Machinery

The stereocilia are located on the apical pole of the hair cells. On the IHCs, they are arranged in a near linear array, while on the OHCs the array is W-formed. The rigid stereocilia are composed primarily of actin and are crosslinked, stimulated by nanometer displacements and graded in height from the shortest to the longest (Flock and Cheung, 1977; Tilney et al., 1992). There are three different types of crosslinks: 1) those that run laterally along each row joining the stereocilia of the same row, allowing the stereocilia of a row move when some have been deflected; 2) those that run laterally between the rows that hold the tips of the shorter stereocilia in toward the taller neighbor; and 3) one per shorter stereocilium running upward toward its taller neighbor that is involved in mechanotransdution (MET) (Pickles et al., 1984). The tip links become stretched when the stereocilia bundle is deflected. In bullfrog hair cells, the movement of the stereocilia toward the kinocilium has been shown to result in depolarization, whereas deflection in the opposite direction causes hyperpolarization (Hudspeth and Corey, 1977). Similar findings were found in mammalian species where deflections toward the longest stereocilium resulted in depolarization (Géléoc et al., 1997; Kennedy et al., 2003; He et al., 2004). The stereocilia pivot at their insertion points at the level of the cuticular plate, causing mechanical forces to open the MET channel (Strelioff and Flock, 1984; Crawford and Fettiplace, 1985; Howard and Ashmore, 1986; Howard and Hudspeth, 1988). It is estimated that there is one (Géléoc et al., 1997) to two (Beurg et al., 2009; Fettiplace et al., 2022) transduction channel for each tip link.

Knowledge of the molecular composition of the MET channel assembly largely stems from genetic studies on monogenic forms of deafness, dominant or recessive, that have provided a number of candidate genes (Richardson et al., 2011), that have then been back-translated and validated in animal models. The tip links are formed from heterodimers of the transmembrane proteins protocadherin-15, forming the lower one-third of the tip link, and cadherin-23, forming the upper two-thirds of the tip link (Kazmierczak et al., 2007). Also found at the upper end of the tip link are myosin motors (myosins IC and VIIA) (Pan and Holt, 2015; Zhao and Müller, 2015), and other proteins such as USH1G, USH, and harmonin (Grati and Kachar, 2011). The channel complex at the lower end of the tip link includes the molecules TMC1, TMC2, LHFPL5, TMIE, TOMT, and CIB2 (Kurima et al., 2002; Santos et al., 2006; Du et al., 2008; Pan et al., 2013; Giese et al., 2017; Cunningham et al., 2020; Jia et al., 2020; Zheng and Holt, 2021).

### B. Cochlear Innervation

Cochlear innervation by SGNs is composed of 95% type I auditory afferent nerve fibers and 5% type II nerve fibers. Type I nerve fibers are myelinated and thick; they are mainly connected to IHCs and send sound information to the brain (Spoendlin, 1969, 1972). Type II afferent fibers are unipolar, unmyelinated, and relatively thin, and each one extends to more than 10 OHCs (Ginzberg and Morest, 1983; Berglund and Ryugo, 1987). In contrast, OHCs receive only 5% of the afferent innervation. Single-cell RNA sequencing (scRNAseq) studies show that the types I and type II afferent fibers are also distinguishable molecularly with more than 1700 differentially expressed genes, 350 of which are clearly binary in their expression, with type Is expressing very specific markers such as *Epha4, Kcna1, Calb2,* and *Pvalb* and type IIs expressing markers such as *Prph, Plk5, Th,* and *Cacna1g,* among others (Sun et al., 2018; Petitpré et al., 2018; Shrestha et al., 2018).

Each IHC is innervated by approximately 20 nerve fibers, depending on cochlear location (Liberman, 1980). Upon stimulation, the IHCs use the synchronized release of hundreds of synaptic vesicles in a manner graded with sound intensity (Glowatzki and Fuchs, 2002). Electrophysiological studies have attempted to record from the type II fibers, but this has proven difficult because of the low numbers and small diameters of their axons (Brown, 1994; Robertson et al., 1999). Compared with the type I synapse, the OHCs have fewer vesicles and a reduced vesicle release probability (Weisz et al., 2014). A study by Robertson et al. in a guinea pig demonstrated that the type II fibers responded to loud sound (Robertson, 1984). Type II neurons also respond weakly to glutamate release from OHCs and more strongly to ATP release (Weisz et al., 2009), most likely from surrounding supporting cells (Lahne and Gale, 2008). The function of the type II SGNs is not well understood, but one current hypothesis is that type II afferent activation by loud sound and modulation by ATP from supporting cells is similar to nociception (thus referred to as auditory nocioception) to protect the hair cells from high sound levels (Flores et al., 2015; Liu et al., 2015).

Type 1 SGNs have three subtypes, and these are classified according to morphological and physiological traits such as threshold and spontaneous firing rate (SR) (Liberman, 1978, 1982; Taberner and Liberman, 2005). The type with low thresholds and high spontaneous firing rates (high SR) respond to low-intensity sounds. Conversely, the subtype combining high thresholds and low spontaneous rates (low SR) can detect high-intensity stimuli. The third subtype displays a combination of low and high SR fibers. Further molecular characterization of SGN using scRNAseq confirmed three transcriptionally unique type 1 subtypes (Sun et al., 2018; Petitpré et al., 2018; Shrestha et al., 2018). The molecular identity of type I neuron subtypes also displays unique profiles but more often a combination or gradients of markers. It is believed that these three subtypes correspond to the thresholds and spontaneous activity of the nerve fibers described previously. Type 1 A would correspond to the high SR expressing high levels of *Calb2* and *Pcdh20*, type 1B the medium SR expressing high levels of *Calb1* and *Lrrc52,* and type 1C the low SR expressing *Pou4f1* and *Lypd1* (Sun et al., 2018; Petitpré et al., 2018; Shrestha et al., 2018). These findings illustrate the complexity of SGN molecular signatures and their correlation with their functional characterization but inform on potential markers that can be used for the selective pharmacological targeting or development of neurons implicated in specific forms of hearing loss.

Both the IHCs and OHCs receive efferent innervation originating in the superior olivary complex (Guinan et al., 1983, 1984). The base of the OHCs receives innervation from the medial olivocochlear efferent neurons (Warr and Guinan, 1979) that release acetylcholine to activate *α*9/*α*10 ACh receptors that allow Ca^2+^ to enter the OHCs, which activates nearby Ca^2+^-dependent K^+^ channels that allow K^+^ to flow out of the cell and causes hyperpolarization. The functional consequence of medial olivocochlear activation is to hyperpolarize the OHCs that shunt depolarization from MET, resulting in reduced somatic motility and amplification. The IHCs have lateral olivocochlear (LOC) efferent fibers that synapse on the unmyelinated dendrites of type I SGNs. The LOC originates in the region surrounding the lateral superior olive. The LOC releases several transmitters, including dopamine, GABA, acetylcholine, and peptides (Niu and Canlon, 2006; Ruel et al., 2007). Darrow et al. (2006) demonstrated two primary types of LOC synapses based on firing patterns that release acetylcholine and dopamine, respectively (Darrow et al., 2006). However, it is unknown whether these two synapses have opposing actions on the afferent auditory nerve fibers. One primary function of the LOC is to reduce damage to the afferent auditory nerve fibers from excessive sound stimulation (Kujawa and Liberman, 2009).

### C. The Cochlear Powerhouse—The Stria Vascularis

The lateral wall of the scala media contains three structures: the outer spiral sulcus, the stria vascularis (SV), and the spiral ligament. The outer spiral sulcus has two cell types, the Claudius cells and the outer sulcus cells, or root cells. Both cell types are believed to be involved in maintaining cochlear fluid balance by recycling potassium for the homeostasis of the high K^+^ levels (∼157 mM) in endolymph (Kikuchi et al., 1995). The functions may differ between root cells in the apical and basal portions of the cochlea, as root cells in the apex express the water channel protein, aquaporin 5, while aquaporin 4 is expressed at the basal portion of the outer sulcus (Eckhard et al., 2015). The cells in the outer sulus are essential to the recycling of K+ ions that maintain the ion content in the endolymph as well as the endocochlear potential (EP) (Kikuchi et al., 2000; Wangemann, 2006). Potassium ions are then released through basolateral K+ channels and subsequently taken up by the supporting cells.

**Fig. 1:**
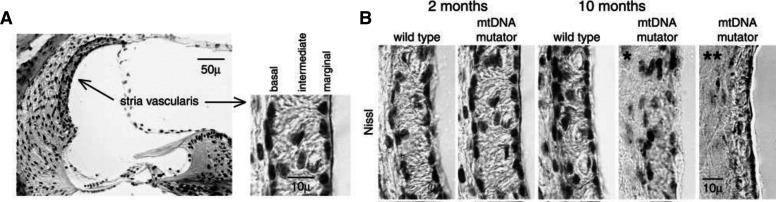
Histology of the stria vascularis and effects of aging. (A) Light microscopic image showing a cross-section through the OC illustrating the lateral wall that includes the SV and the spiral ligament. The SV contains three cell layers: the basal, the intermediate, and the marginal cells. The marginal cells face the endolymph. (B) Sections stained for Nissl (cresyl-violet) illustrate the SV from wild-type and a model for aging, the mitochondrial DNA mutator mice at 2 and 10 months of age. All sections are obtained from the middle turn of the cochlea. The SV from the 10-month-old mitochondrial DNA mutator mice demonstrate a disorganized (*) and a thinning (**) of the SV (Niu et al., 2007).

The SV is a highly vascular tissue in the lateral wall and has three major cell types (marginal, intermediate, and basal cells) along with a minor number of spindle cells, pericytes, and endothelial cells (Fig. 1). Together, these cells maintain the ionic composition of the endolymph and produce the EP (Salt et al., 1987; Wangemann, 2006; Nin et al., 2016). The EP has a direct current potential of +80 mV that drives the K+ ions of the endolymph through the transduction channel at the apical pole of the hair cells. For this reason, the SV is often referred to as the cochlear battery, or the powerhouse.

A number of genetic mutations have been associated with dysfunctions of the SV that cause hearing loss (Zhang et al., 2020). These mutations can target proteins located in one or more of the three cell types (i.e., basal, intermediate, or marginal cells) and affect one key step in the transport of K^+^ from the blood to the scala media. The *marginal cells* are in contact with the endolymph and are involved in the transport of K+ ions (Wangemann, 1995; Kim and Ricci, 2022). In particular, the K+ channels in the intermediate cells are essential to K+ transport, which regulates the level of the EP. The *intermediate cells*, which are melanocytes, lie between the marginal and basal cells. Pathology changes of the intermediate cells result in a diminished EP (Steel and Barkway, 1989), since the intermediate cells regulate the K+ concentration and transport through the SV (Chen and Zhao, 2014). The *basal cells* are connected via gap junctions to the fibrocytes of the spiral ligament and recycle K+ from the perilymph to maintain the EP. Claudin 11 is one of the proteins that is highly specific to a single cell type and for which animal mutants exist. Claudin 11 is located in basal cells, and its dysfunction causes profound hearing loss in mice, associated with a loss of the EP (Gow et al., 2004). Evidently, maintenance of the EP is complex and involves the contribution of the different SV cell types, which appear to play an important role in normal hearing. One mouse model of aging, the mitochondrial mutator mouse (mitochondrial DNA) (Niu et al., 2007), shows degeneration of the different SV cell types, with aging correlating with increased auditory thresholds (Fig. 1).

Due to the difficulty in accessing the human cochlea, most of our knowledge stems from experimental research on animals. ATP6V0A4 causes distal renal tubular acidosis and sensorineural hearing loss in humans. Mice that lack ATP6V0A4 expressed in intermediate cells are completely deaf and also lack the EP (Lorente-Cánovas et al., 2012). Marginal cells, by contrast, have several more mouse models deriving from human syndromes. For example, loss of function of the KCNQ1 or the KCNE1 subunit of the apical K+ channel of marginal cells causes deafness in mice and humans (Vetter et al., 1996), as does the disruption of their basolateral NaK2Cl cotransporter in mice (Delpire et al., 1999). Likewise, human Bartter syndrome IV is an autosomal recessive disorder characterized by congenital deafness and severe renal salt and fluid loss. It is caused by mutations in BSND, which encodes Barttin, a *β*-subunit of ClC-Ka and ClC-Kb chloride channels. Inner-ear-specific disruption of *Bsnd* in mice reveals that the endocochlear potential, but not the high potassium concentration, of the scala media depends on the presence of these channels in the epithelium of the SV (Rickheit et al., 2008).

## III. Delivery Routes to the Cochlea

**Fig. 2:**
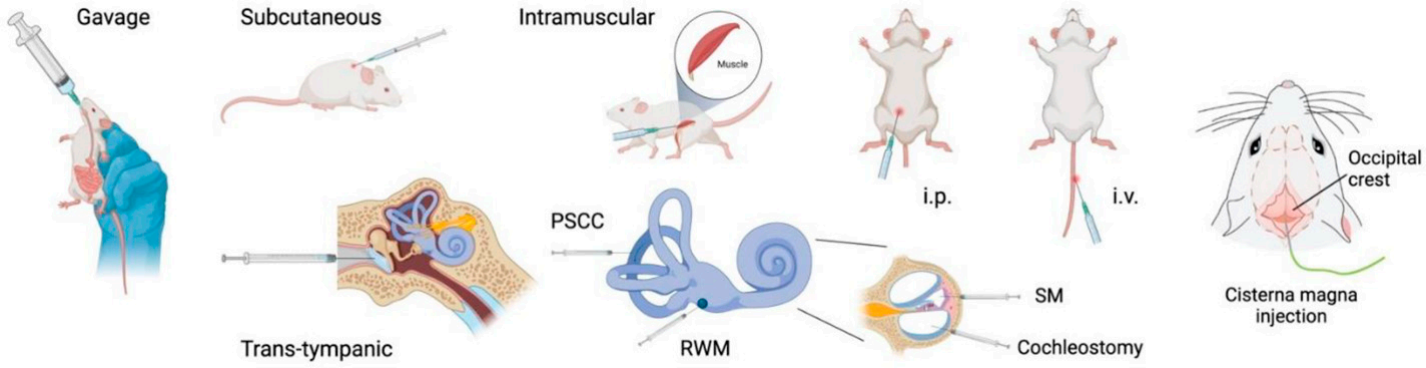
Drug delivery routes in preclinical hearing research. Illustrating the most common procedures for delivering drugs to the inner ear. ip, intraperitoneal; iv, intravenous; PSCC, posterior semicircular canal; RWM, round window membrane; SM, scala media.

When pharmacological agents are being delivered, it is imperative to maximize their therapeutic efficacy. This can be achieved by ensuring that target exposure is sufficient to obtain the desired benefit while also minimizing risk of adverse events from off-target effects, depending on the properties of the pharmacological agent. The many delivery procedures that are commonly used in the preclinical auditory field include standard systemic routes (intravenous, intraperitoneal, subcutaneous, intramuscular oral, and nasal administration) as well as approaches that leverage more direct delivery into the fluid compartments of the inner ear (Fig. 2).

### A. Systemic Approaches and the Blood-Labyrinth Barrier

With systemic delivery, a critical question arises concerning the distribution of a drug to the inner ear compared with other compartments in the body. Some studies have used induced-coupled plasma mass spectrometry to measure ototoxic drug levels in the cochlea after intraperitoneal injections (Breglio et al., 2017; Tserga et al., 2020) or determined the distribution of dexamethasone to the perilymph after intravenous or intraperitoneal injection (Wang et al., 2018; Ke et al., 2023) or the dose-dependent target exposure of novel otoprotective drug candidates after oral administration (Petremann et al., 2017) using both liquid chromatography and tandem mass spectrometry. However, such approaches are not routinely included in most auditory research efforts involving systemic drug administration. Although systemic delivery is clearly capable of achieving inner ear drug exposure for compounds with appropriate individual characteristics, it typically leads to general exposures in other compartments and may thus increase the risk of side effects, depending on the drug candidate profile.

The systemic delivery of substances aimed at targeting the inner ear needs to bypass the blood-labyrinth barrier (BLB), which divides the vasculature and the perilymph and endolymph. The BLB is fully developed by postnatal day 14 (Suzuki and Kaga, 1999). Its primary function is to maintain the ionic homeostasis of the inner ear fluids and protect the inner ear from substances that have potential adverse effects (Shi, 2011). It is composed of tightly coupled vascular endothelial cells, perivascular resident macrophage-like melanocytes, and pericytes and fibrocytes in the lateral wall (Zhang et al., 2012; Neng et al., 2013). When treating the inner ear with pharmacological substances, it is important to know whether the drug in question can pass this barrier and achieve target exposure. The tight junctions making up the BLB limit the entry of molecules or drugs to the inner ear.

The BLB may limit the accessibility of specific systemically delivered drugs to the inner ear and reduce their therapeutic efficacy, specifically in the case of repurposed drugs that were not initially developed to target the inner ear. Formulation efforts have been made to improve the passage through the BLB and thus enhance distribution into the inner ear. This includes the addition of lipid molecules to increase the hydrophilic nature of the substance. Juhn and Rybak suggested that intravenously injected substances are transported into the cerebrospinal fluid (CSF) and the perilymph via different mechanisms (Juhn and Rybak, 1981; Juhn et al., 1981). The permeability of the BLB can be disrupted by osmotic agents (including diuretics), inflammation, and traumatic noise exposure (Shi, 2016). For instance, the diuretic furosemide can increase the entry of cisplatin and aminoglycosides into the cochlea by disrupting BLB function (Mulders et al., 2014; Li and Steyger, 2011). Acoustic trauma can also increase the permeability of the BLB by damaging the tight junctions (Wu et al., 2017; Ke et al., 2023).

The brain is protected by systemically delivered drugs and toxins by the blood-brain barrier (BBB), which has certain similarities to the BLB. The main function of both the BLB and the BBB is to separate the blood from interstitial fluid to maintain homeostasis. After an intravenous injection of a radioactive tracer, the BLB has been shown to be less permeable than the blood-CSF barrier. After 90 minutes, more of the tracer was in the CSF compared with in the perilymph (Inamura and Salt, 1992). However, the aminoglycoside antibiotic gentamicin can cross the BLB but not the BBB, indicating that these differences could be due to the tightness of the barrier or transport mechanisms over the barrier (Neuwelt et al., 1984). For more information on the differences between the BLB and the BBB see Nyberg et al., (2019).

### B. Local Delivery of Drugs

To overcome target exposure difficulties or safety concerns for specific drug candidates, investigators have sought more controlled and reliable routes of delivery to the inner ear. Several procedures can be used to locally deliver drugs to the inner ear, with varying degrees of risks for causing damage to the inner ear. The first approach is to inject the drugs through the tympanic membrane. This is commonly used in the clinic. It is also used on experimental animals where, after being infused into the middle ear, the drug is expected to diffuse across the round window membrane (RWM) and into the perilymph. The RWM is a barrier between the middle ear and the scala tympani. It is semipermeable and allows the passage of numerous substances, depending on their molecular size, concentration, and electrical charge (Goycoolea, 2001). Adding excipients such as DMSO, saponin, or benzyl alcohol can improve penetration through the RWM (Li et al., 2018). Likewise, the use of biodegradable hydrogels placed near the RWM improves cochlear exposure (Endo et al., 2005). Another approach includes intratympanic injection through the otic bone into the middle ear which does not compromise the tympanic membrane (Oishi et al., 2013).

The second approach for improving the control and reliability of drug delivery to the inner ear includes intracochlear procedures. Despite requiring invasive surgery and increasing the risk of damaging the cochlea, these procedures are commonly used in experimental animals. For instance, delivery through the RWM is known to increase drug concentration in the inner ear when compared with systemic injections. However, this procedure can cause damage to the membrane itself and decrease perilymphatic pressure (Plontke et al., 2016; Szeto et al., 2020). There is also the risk of inserting the injection needle too far in and damaging the basal turn of the cochlea. New techniques such as microperforations to the RWM can improve permeability and reduce the risk of damage (Kelso et al., 2015). A third approach is to injection into the semicircular canal (Salt et al., 2012). Using the semicircular canal has the advantage of efficiently introducing drugs or viral vectors into the inner ear without compromising hearing thresholds.

### C. Cerebrospinal Fluids Linked to the Cochlear Perilymph

A more recent approach, possibly an intermediary method between systemic and local injections, has been to use the cisternae magna (CM) as an injection site (Fig. 2). The cisterna magna is located in the posterior fossa, dorsal to the medulla and caudal to the cerebellum. It contains CSF, which is transported along the perivascular spaces in what has been termed the glymphatic system (Iliff et al., 2012). Glymphatic fluid transport plays an important homeostatic role, as fluid efflux clears metabolic waste products from the brain (Lohela et al., 2022). The CM freely communicates with the subarachnoid space, which connects the cerebrospinal fluid to the perilymph of the scala tympani via the cochlear aqueduct. Large-particle tracers (e.g., gadobutrol 0.6 kDa) injected into the CM reach the inner ear through dispersive transport via the cochlear aqueduct in adult mice within minutes (Mathiesen et al., 2023). Amine-modified polystyrene microspheres (0.2 and 1 *μ*m in diameter), which could be used for drug delivery, can also reach the cochlea through this route (Mathiesen et al., 2023).

Connections between the inner ear fluids and the CSF have previously been suspected, particularly because the delivery of adeno-associated viruses (AAVs) through the RWM can reach the contralateral ear (Stöver et al., 2000). Recently, AAVs injected in the CM were shown to reach and transduce both left and right cochleae (Blanc et al., 2021). The therapeutic potential of this route was demonstrated by Koch-Mathiesen et al., who showed that in adult deaf *Slc17A8 -/-* mice, a single CM injection of a vesicular glutamate transporter-3 (VGLUT3) expressing AAV can effectively transduce IHCs and fully rescue auditory brainstem responses to levels equivalent to wild-type mice (Mathiesen et al., 2023). A similar connection between CSF and the cochlea has been suggested in the rhesus macaque (*Macaca mulatta*), where intracerebroventricular injections of AAV9.EGFP lead to cochlear IHC transduction together with cells of the spiral ligament, and cells of the spiral limbus (Ranum et al., 2023), supporting the translational potential of the CM route for gene therapy.

The application of this approach in humans has been debated, particularly due to the possibly reduced patency of the cochlea aqueduct, which is thought to decline with age (Włodyka, 1978; Gopen et al., 1997). Indirect measures of intracochlear pressure changes during postural changes revealed that the aqueduct was functionally patent in 89% of young adults and in 70% of older adults (Phillips and Marchbanks, 1989; Wagner and Walsted, 2000). Several studies suggest that the cochlear aqueduct in humans can transfer intracranial pressure changes and that enlarged intracranial pressure can be detected by measuring intratympanic pressure (Shimbles et al., 2005; Gwer et al., 2013; Evensen et al., 2018). Interestingly, when assessing magnetic resonance imaging data from cisternograms performed on individuals to assess CSF leaks at the base of the skull, Totten et al. observed the progressive diffusion of gadolinium contrast into the human cochleae and vestibule (Totten et al., 2023). Several ongoing trials are using cisternae magna injections for neurodegenerative diseases (Taghian et al., 2020; Marchi et al., 2022). Overall, these findings strongly support that cerebrospinal fluid transport serves as an effective and accessible route for gene delivery or other otoprotective agents to repair the adult inner ear and thus represents a crucial step toward restoring hearing in rodents and humans.

## IV. Structural Damage and Molecular Pathways in the Cochlea: From Noise and Aging to Ototoxic Drugs

**Fig. 3:**
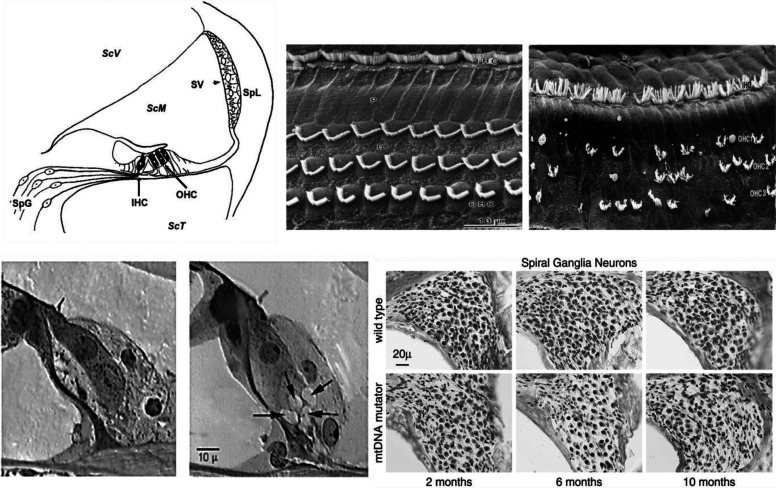
Anatomical and histological alterations upon cochlear damage. Examples of the alterations that can occur after damage to the cochlea. (A) Schematic cross-section of the OC showing the inner hair cells (IHCs) and outer hair cells (OHCs), the stria vascularis (SV), the spiral ligament (Spl), scala media (ScM), SGNs (SpG), and the two scala containing perilymph Scala vestibuli (ScV) and Scala tympani (ScT). (B) Scanning electron microscopy images of the apical surface of the OC showing the normal (left) array of stereocilia on the IHCs (upper portion of image) and the three rows of OHCs; (right) an example after noise trauma showing disarrayed IHC stereocilia and missing and disarrayed OHC stereocilia. (C) Section showing the IHCs with intact afferent dendrites at the basal end of the cell and on the right, an example of excitotoxicity after trauma. The arrows are pointing to swollen afferent dendrites. (D) Progressive loss of SGNs in a mouse model of aging, the mitochondrial DNA mutator with increasing age. Representative micrographs showing bundles of SGNs from the middle turn of the wild-type and the mitochondrial DNA mutator mice at 2, 6, and 10 months of age.

Damage to the cochlea has various causes including noise trauma, aging, and ototoxic drugs. These insults can affect the sensory cells and neurons and the nonsensory structures in the cochlea, which can result in reduced hearing sensitivity of various degrees (Fig. 3).

### A. Noise Trauma

Noise exposure can cause either temporary or permanent hearing loss. In general, temporary hearing loss occurs when the intensity is low to moderate, whereas a permanent hearing loss results from higher intensities. When the intensity of the exposure is high, a combination of mechanical damage and metabolic stress occurs in the cochlea, causing either OHC loss, OHC and IHC loss, or complete loss of the organ of Corti (OC). The damage is irreversible since sensory cells do not spontaneously regenerate. OHCs are more susceptible to cell death than are IHCs. When an OHC dies and degenerates, the hole is then replaced by a scar formed by neighboring phalangeal cells (Raphael et al., 1993). Damage to the stereocilia on the apical portion of the hair cells can include detachments from the tectorial membrane, breaks in the tip links, and loss of rigidity, making them unable to transduce mechanical energy to the cell body (Liberman and Dodds, 1987; Jia et al., 2009). The stereocilia, however, have the potential to repair themselves (Wagner and Shin, 2019), a potential mechanism underlying temporary noise-induced hearing loss. Noise trauma can permanently affect the afferent synapse of the IHCs due to an excessive release of glutamate, which causes excitotoxicity (Puel et al., 1998). Different types of supporting cells in the OC can also show pathology after noise trauma; however, these changes are typically found after impulse noise or very high-intensity sounds (Nordmann et al., 2000) and can depend on genetic background (Herranen et al., 2018).

Noise overstimulation can produce hearing loss in which OHCs, particularly those at the high-frequency region of the cochlea, are affected more than at the low frequency region. It has been suggested that the differential vulnerability can be due to differences in the Ca2+ balance among cochlear locations. Intracellular Ca2+ homeostasis is determined by Ca2+ influx through the mechanotransducer channels and efflux by the plasma membrane CaATPase pump. As a result of noise overstimulation, a Ca2+ overload is thought to make the OHCs in the high-frequency region more vulnerable to noise overstimulation (Fettiplace and Nam, 2019). A third type of hearing loss induced by noise trauma is referred to as hidden hearing loss. This type of hearing loss is distinguished by a loss of the synaptic connections from the hair cell to the neurons, without any hair cell loss (Furman et al., 2013). In mice, a permanent reduction in the number of ribbon synapses after noise trauma is a feature of hidden hearing loss reflected by a decrease in the amplitude from the wave 1 of the auditory brainstem response (ABR) without evidence of a permanent threshold shift (Lin et al., 2011). The ABR wave I amplitude is a measure of auditory nerve activity and noise trauma can cause cochlear deafferentation and reductions in wave 1 amplitude (Kujawa and Liberman, 2009). The functional consequence of this selective loss would lead to difficulties in understanding speech, particularly in noisy environments (Oxenham, 2016; Bakay et al., 2018). These noise-induced changes can eventually lead to a loss of SGNs (Kujawa and Liberman, 2006).

As mentioned above, the SV is also a generator of the endolymphatic potential, which is essential for sensory hair cell transduction and contains marginal, intermediate, and basal cells. The intrastrial fluid-blood barrier contains perivascular resident macrophages that are required for maintaining the EP potential and keeping the tight junctions in the barrier. Acoustic trauma has been shown to break down the tight junctions, resulting in the leakage of serum proteins into the endolymph (Zhang et al., 2013). These changes will affect the endolymphatic potential and result in reduced hearing sensitivity.

### B. Aging

Presbycusis or age-induced hearing loss is a complex phenomenon due to the multitude of factors that can influence the hearing organ. Such factors include prior noise exposure or exposure to ototoxic drugs or solvents, genetic factors, vascular pathology, infection, or other hearing related disorders (Keithley, 2020; Eckert et al., 2021). These different factors that cause cell degeneration and hearing loss result from oxidative stress, mitochondrial DNA damage, inflammatory processes, and impaired vascular supply to the inner ear (Seidman et al., 2002; Watson et al., 2017).

Through aging, the OHCs are more susceptible to cell death compared with the IHCs in both human and rodent cochlea (Bredberg, 1968; Wu et al., 2019). In humans, the loss occurs in the apical and basal regions and progresses to the other regions of the cochlea. In rodents, the loss of OHCs starts at the basal region of the cochlea and proceeds toward the apex. Spiral ganglion loss is also apparent in the human and rodent inner ear. During aging, neuronal loss has been demonstrated to be greater than IHC loss (Viana et al., 2015). This is an interesting observation and suggests that, during aging, the SGNs are disconnected from the IHCs.

Over the years, the SV has been indicated as a significant contributor to age-induced hearing loss—a finding that has also been recently supported by genetic studies in humans (see Section VI.). Damage to the lateral wall will alter cochlear homeostasis, potassium recycling, and the maintenance of the EP that drives current through the hair cells and maintains normal hearing. Raising gerbils in a quiet environment revealed that aged gerbils display a significant degree of degeneration of the lateral wall and reductions in the EP (Gratton et al., 1996). The mitochondrial DNA mutator mouse also shows increasing degeneration of the cells in the SV with age (Fig. 1).

In the spiral ligament, type IV fibrocytes show pathological changes with increasing age in the human cochlea, a phenomenon that has been also found in aged gerbils and mice (Spicer and Schulte, 2002; Wu and Marcus, 2003). Surprisingly, spiral ligament fibrocytes degenerate before any loss of hair cells occurs. In animal studies, it appears that age-related hearing loss (ARHL) is the result of strial atrophy and fibrocyte degeneration in the spiral ligament. Earlier studies of human presbycusis suggested that strial atrophy or degeneration of auditory nerve fibers rather than hair loss was the cause of the age-induced hearing (Schuknecht, 1964; Schuknecht and Gacek, 1993). In a more recent study, an analysis of autopsy specimens showed that hair cell degeneration outweighed the effects of strial atrophy (Kaur et al., 2023). These distinctions may be a reflection of species differences as well as the histological procedures that were used in the early human studies.

Another morphological feature that can explain part of the hearing loss induced by age is the myelination of the type 1 SGNs. Myelination of the auditory nerve is required for normal auditory nerve function and the transmission of electrical impulses from the cochlea to the auditory brain (Wan and Corfas, 2017). Degradation of the myelin on nerve fibers and reductions in myelin basic protein have been found in aged animals (Cohen et al., 1990; Xing et al., 2012) and in older human samples. These findings suggest that the degeneration of the myelin sheath around nerve fibers can alter hearing sensitivity in aged subjects.

### C. Ototoxicity

Ototoxicity is the process by which drugs and medication negatively impact the function of the inner ear. More than 100 different types of drugs have been found to be ototoxic, including anticancer drugs, aminoglycoside and nonaminoglycoside antibiotics, and antimalarial drugs, leading to an estimated 20 million new cases of hearing loss worldwide per year (Prasad et al., 2024). This section will focus only on the impact of cisplatin and aminoglycosides on hearing.

#### 1. Cisplatin

Cisplatin (cis-diammine dichloroplatinum II) is a highly effective chemotherapeutic agent commonly used in the treatment of solid tumors and several types of malignant tumors, such as those of the head and the neck, in adults and children (Wang and Lippard, 2005; Dasari and Tchounwou, 2014a). Cisplatin is known to have numerous debilitating side effects such as permanent hearing loss, neurotoxicity, and nephrotoxicity (Dasari and Tchounwou, 2014b; Sheth et al., 2017). Carboplatin is also a platinum-based drug used against cancer but has less toxicity than cisplatin. The prevalence of hearing loss following cisplatin treatment is dependent upon the cumulative dose and can range from 4% to 90% depending on a variety of factors such as the age and gender of the patient, which combination of other ototoxic agent(s) or irradiation are used, exposure to concomitant noise, duration of the treatment, and treatment administration methods (Karasawa and Steyger, 2015; Landier, 2016). A recent systematic review of 66 studies has estimated that the global prevalence of ototoxic-induced hearing loss is 43.2% (Dillard et al., 2022). This study revealed that the prevalence of hearing loss for cisplatin alone was 49.2%, whereas that of carboplatin alone was 13.5%. Treatment regimens involving both cisplatin and carboplatin increased the prevalence to 73.2% in children under 5 years of age. One major obstacle to estimating the true impact of platinum-based therapy in humans is the method used to assess hearing loss, which ranges from audiometry to OAEs and other ototoxicity grading scales (e.g., ASHA, CTCAE, Brock, Chang), some of which are exclusively designed for pediatric use. Nonetheless, the prevalence estimates are relatively similar across these different diagnostic methods and imply that nearly half a million cases of hearing loss per year occur based on ototoxicity.

Cisplatin-induced hearing loss typically affects the high-frequency regions at the base of the cochlea and eventually affects the lower frequencies with continued use. In experimental animals, the key structures affected by cisplatin resulting in hearing loss include the marginal cells of the SV, the spiral ligament, and the OHCs; and with extended use, the IHCs, supporting cells, and the SGNs are also affected (Breglio et al., 2017). Similar to the actions of aminoglycosides, cisplatin also enters the inner ear through the bloodstream and must pass the BLB. Cisplatin enters the inner ear fluids via the SV and then enters the hair cells (Prayuenyong et al., 2021). One entry route of cisplatin to the hair cells is via the mechanosensitive transduction channels. Use of fluorescently tagged cisplatin revealed that cisplatin enters the hair cells through mechanoelectrical transduction channels at the tip of the stereocilia (Waissbluth and Daniel, 2013; Kros and Steyger, 2019; Thomas et al., 2013). Moreover, the prolonged effects of cisplatin in the cochlear tissues cause further pathology to the different cell types. This is because platinum remains in the cochlea for long periods of time (Breglio et al., 2017).

Molecular pathways that involve cisplatin-induced ototoxicity include reactive oxygen species (ROS) generation, inflammation, and autophagy, ultimately resulting in cell death. The underlying basis of hearing loss due to ototoxic medications is multifactorial since the drugs can impact the production of the EP, the function of OHCs and their survival, and the coupling between the IHC-afferent neuron synapse. Cisplatin can also enter hair cells by both passive diffusion and active uptake. IHC uptake involves copper transporters such as CTR1 and organic cation transporters such as OCT2. Once in the cells, cisplatin can be hydrolyzed by water to generate aqua-cisplatin complexes that are toxic and can damage DNA. This damage leads to the upregulation of ataxia telangiectasia mutated, which activates the tumor suppressor p53 and leads to the release of the proapoptotic protein Bcl-associated X, ultimately increasing mitochondrial membrane permeability and releasing cytochrome c via caspase 3 activation (Wang et al., 2004; Benkafadar et al., 2017). Entry into hair cells may also occur through the MET, since the blockade of MET channels can reduce cisplatin ototoxicity (Thomas et al., 2013). Hair cells have a high metabolic rate since they have to process information with high spectral and temporal precision of up to 20 kHz in humans and 60 kHz in rodents. Such systems require an active antioxidant defense mechanism to regulate ROS, when subjected to noise or ototoxicity. Some NADPH oxidases (NOX), such as NOX3, are highly abundant in the inner ear and are strongly induced by cisplatin (Mukherjea et al., 2010). Their blockade using small interfering RNA administration in the middle ear cavity can attenuate cisplatin-induced hearing loss.

Oxidative stress occurring in hair cells exposed to cisplatin decreases the levels of antioxidant enzymes such as superoxide dismutase, glutathione reductase, glutathione S-transferase, and glutathione peroxidase (Rybak et al., 2000; Campbell et al., 2003). This ultimately leads to the release of cytochrome c and apoptosis. Cisplatin-mediated hair cell death involves not only apoptosis but also ferroptosis and necroptosis. Ferroptosis involves lipid peroxidation, iron accumulation, and the reduction of the mitochondrial membrane potential, all of which precede autophagy. Necroptosis is a passive form of necrosis mediated by receptor interacting protein kinases. Intraperitoneal injection of Necrostatin-1 s, an inhibitor of receptor interacting protein kinase 1, protects against cisplatin-mediated hearing loss in rats (Choi et al., 2019). Downstream of lipid peroxidation is the generation of toxic reactive aldehydes. These reactive aldehydes can activate nonselective cation permeable channels such as the transient receptor potential (TRP) channels, which are involved in taste, touch, smell, and pain. TRPV1 is one type of TRP channel identified in the cochlea that is activated by NOX3-mediated release of ROS. Transtympanic administration of TRPV1 small interfering RNA protects against cisplatin-mediated hearing loss (Mukherjea et al., 2008).

#### 2. Aminoglycosides

Aminoglycoside antibiotics (e.g., kanamycin, neomycin, amikacin, capreomycin, or streptomycin) are used against multidrug-resistant tuberculosis. They are therefore likely to be the most commonly used antibiotics worldwide. A meta-analysis of 18 studies from 10 countries has estimated a global prevalence of aminoglycoside-mediated hearing loss of 40.6% for all drugs (Dillard et al., 2021). Individually, kanamycin reached the highest prevalence (49.7%), followed by amikacin (38.9%) and capreomycin (10.2%). Similar to cisplatin-induced ototoxicity, aminoglycoside ototoxicity can potentially result in the hearing loss of nearly half a million individuals with drug-resistant tuberculosis. The application of World Health Organization guidelines may prevent 50 million cases of hearing loss per year (Dillard et al., 2021).

Aminoglycosides enter through the bloodstream and through the BLB, a specialized structure consisting of tight junction-coupled inner ear endothelial cells, which separates the inner ear tissues from the bloodstream (Li and Steyger, 2011). Aminoglycosides then pass through the different cell types in the SV—first the basal, then the intermediate, and finally the marginal cells. The aminoglycosides primarily enter the endolymph, which is the source of uptake into the hair cells (Li and Steyger, 2011). Electrophysiological studies show that aminoglycoside uptake into the hair cells occurs through mechanosensitive transduction channels (Marcotti et al., 2016) and that functional mechanotransduction channels are required for ototoxicity (Alharazneh et al., 2011). The OHCs are more susceptible to aminoglycosides than are the IHCs. However, with increasing administration, the IHCs and apical OHCs become damaged. The OHCs die and are replaced by a scar formed by neighboring phalangeal cells. Synaptopathy is also evident in cochleae exposed to aminoglycoside antibiotics, where loss of IHC synapses appears before threshold shifts are apparent (Ruan et al., 2014). Ototoxicity starts in the high-frequency region and with extended use the damage progresses to the lower-frequency range (Zettner and Gleser, 2018). The hearing loss induced by aminoglycosides starts in the basal, high-frequency region and with prolonged use will spread to the more apical regions of the cochlea.

Mechanisms regulating the vulnerability to aminoglycoside ototoxicity appear to be centered around the mitochondria. A number of variants at the 12S rRNA of the mitochondrial genome are associated with an increased risk of aminoglycoside ototoxicity (e.g., A1555G, 745 A > G, 792C > T, 839 A > G, 856 A > G, 1310C > T, and 1452T > C), making the 12S tertiary structure more similar to the bacterial 16S rRNA and thus a more likely target of aminoglycosides (Prezant et al., 1993; Lu et al., 2010). Similar to cisplatin ototoxicity, aminoglycoside antibiotics have been reported to generate free radicals in the inner ear and to damage sensory cells and neurons. Mice overexpressing superoxide dismutase 1 display less aminoglycoside-induced ototoxicity compared with the wild-type controls (Sha et al., 2001). Indeed, a number of studies have evidenced the protective effects of antioxidants against ototoxicity (Sha et al., 2001; Feldman et al., 2007; Naeem et al., 2009; Kocyigit et al., 2015). Downstream of ROS signaling, aminoglycosides activate the c-Jun N-terminal kinase (JNK), which triggers apoptosis. Delivery of JNK peptide inhibitors via cochleostomy is able to protect guinea pigs from neomycin-mediated ototoxicity (Wang et al., 2003). The mechanisms of aminoglycoside and cisplatin-mediated ototoxicity appear to differ, whereby ROS-JNK pathways causing apoptosis are recruited during aminoglycoside damage but not during cisplatin (Wang et al., 2004), and ROS-Caspase3-p53 signaling pathways are involved in cisplatin-mediated ototoxicity (Wang et al., 2004; Benkafadar et al., 2017).

Seldom are different aminoglycosides and cisplatin compared in terms of molecular mechanisms within a single experimental study. A comprehensive review of the molecular overlaps and points of divergence for the various forms of cochlear damage is beyond the scope of this review, and some aspects have been covered previously (Yang et al., 2015). Since then, there have been multiple advances showing for instance the involvement of the mTOR pathway in noise and cisplatin-induced hearing loss (Fu et al., 2022), as well as epigenetic modifications, which modulation with the inhibitor of the histone H3 lysine 9 dimethylation (H3K9me2) enzyme G9a (BIX01294) can prevent cisplatin-mediated ototoxicity via a miRNA-dependent induction of authophagy (Mu et al., 2023). Mitophagy is the selective degradation of mitochondria (Lemasters, 2005), which includes mitochondrial fission, the marking of specific mitochondria with ubiquitin-dependent or independent receptors, the recruitment of phagophores, and their expansion. The engulfment of mitochondria by autophagosomes and their fusion with lysosomes form the autolysosome for the final degradation of the cell (Montava-Garriga and Ganley, 2020). Although some proteins involved in mitophagy contribute to ARHL in rodents, their role in ototoxicity remains to be established. Recent advances in scRNAseq, which have been used, for instance, to investigate pathways implicated in noise injury in the cochlea (Milon et al., 2021), may increase our knowledge of the molecular mechanisms underlying noise and aminoglycoside- and cisplatin-induced hearing loss.

## V. Drug Treatment in Humans: Evidence from the Ototoxicity Pipeline

Because it is ethically debatable when evaluating the efficacy of a drug to intentionally expose humans to noise in a clinically controlled experimental design, as was the case for ebselen (Kil et al., 2017; Maison and Rauch, 2017), most of the current clinical evidence for protective drugs against hearing loss has emerged from studies on ototoxicity. Only a handful of drugs are currently being tested in humans to reduce cisplatin- or aminoglycoside-induced hearing loss (Lee et al., 2024). Four of these are Food and Drug Administration-approved or repurposed drugs (e.g., sodium thiosulfate, statin modulators, cimetidine, and N-acetylcysteine), and the others are novel developments. The limited pharmaceutical pipeline may stem from the limited translational validation of the preclinical models, the complex clinical development path, or a lack of well-established clinical endpoints and comparators (see Section VII).

Animal studies have shown that sodium thiosulfate (STS) has the ability to inactivate cisplatin by forming platinum thiosulfate complexes that reduce the extracellular levels of free platinum as well as by preventing its cellular uptake. However, since this effect of STS can also impact cisplatin’s antitumor activity when administered simultaneously, timing strategies to administer STS after cisplatin have proven to be beneficial in protecting mice from cisplatin-induced hearing loss without impacting the antineogenic effects of cisplatin (Harned et al., 2008), a beneficial effect on hearing that was replicated in rats and guinea pigs (Muldoon et al., 2000; Dickey et al., 2005). Two completed phase III randomized controlled trials have tested the benefits of intravenous STS in reducing cisplatin-induced hearing loss in pediatric patients (NCT00716976; NCT00652132), both of which showed more than a twofold reduction in the incidence of hearing loss in cisplatin-treated patients when they were given STS (Freyer et al., 2017; Brock et al., 2018). However, the possibility that STS may reduce the survival of pediatric patients with metastatic cancer is currently being evaluated in two other phase III trials (NCT04478292; NCT05382338). Additionally, in two phase II trials (NCT05129748; NCT04541355) are currently evaluating whether STS is also applicable to adults.

Statins have also been suggested as potential protectors to drug-mediated ototoxicity in mice and rats (Fernandez et al., 2020; Lee et al., 2022a). Among patients, statin users have a two- to threefold decrease in the incidence of cisplatin-mediated hearing loss (Fernandez et al., 2021). Two randomized controlled trials are currently testing rosuvastatin (phase II, NCT04817904) or atorvastatin (phase III, NCT04915183) against cisplatin-mediated hearing loss. The low interference of statins with the antitumorigenic actions of cisplatin makes these drugs attractive candidates (Lebo et al., 2018; Gupta et al., 2019).

Knowing the involvement of the antioxidant pathway in both aminoglycoside and cisplatin ototoxicity makes it not so surprising to see Food and Drug Administration drugs, such as *N*-acetylcysteine (NAC), being repurposed in several preclinical studies against the two ototoxic chemical branches (Somdaş et al., 2015, 2020; Chen et al., 2022). One phase II study (NCT01131468) showed outstanding benefits of NAC treatment in protecting against aminoglycoside-induced toxicity in patients with peritonitis resulting from continuous ambulatory peritoneal dialysis (Tokgoz et al., 2011). Regarding cisplatin, there are still uncertainties as to how effective NAC can be, with results from a first trial with oral NAC (NCT02241876) being inconclusive (Visacri et al., 2019). Other trials with either intravenous or intratympanic applications are ongoing, including a phase IV (NCT04226456).

New developments include SPI-1005 from Sound Pharmaceuticals, which is an oral formulation of ebselen that mimics the activity of glutathione peroxidase. Two animal studies have suggested some potential protective effects (Lynch et al., 2005; Kim et al., 2009), which prompted its testing in a phase II trial evaluating oral SPI-1005 against tobramycin in patients with cystic fibrosis (NCT02819856). A promising candidate against aminoglycoside ototoxicity is ORC-13661, which was developed by Oricula Therapeutics based on an initial small-molecule screen in zebrafish with aminoglycosides identifying ORC-001/PROTO-1 (Owens et al., 2008; Chowdhury et al., 2018). ORC-13661 is a chemical optimization of ORC-001, which has demonstrated full protection of the zebrafish hair cells exposed to neomycin and highly effective protection in rats treated with amikacin (Chowdhury et al., 2018). While ORC-13661 will soon be tested in a phase II study (NCT05730283) on patients with nontuberculous mycobacterial infections treated with amikacin, its benefit against cisplatin remains to be demonstrated. Sensorion Pharmaceuticals developed SENS-401 (R-azasetron besylate), a 5-HT_3_ (serotonin) receptor antagonist and calcineurin inhibitor, to block apoptosis during ototoxic damage. Oral administration of SENS-401 protected rats from a single cisplatin intravenous infusion (Petremann et al., 2017). This led to the currently ongoing phase II trial (NCT05628233), which is evaluating the efficacy of SENS-401 against cisplatin ototoxicity in adult patients with cancer. ACOU085, developed by Acousia Therapeutics, is a voltage-gated potassium channel subfamily Q member 4 channel (Kv7.4) activator. Kv7.4 is an important ion channel for OHC survival (Nouvian et al., 2003; Kharkovets et al., 2006), and ACOU085 was shown to protect against cisplatin-mediated OHC loss (Dyhrfjeld-Johnsen, personal communication). A phase IIa clinical trial was recently begun in Europe (EudraCT 2023-503696-15-00).

## VI. The Emerging Translational Evidence: Linking Rodent Molecular Biology to Human Genetics

Various scRNAseq technologies have indeed allowed for a greater resolution in the molecular understanding of the complex anatomical and cellular landscape that characterizes the cochlea. The use of such technologies in animal experiments such as noise trauma have helped identifying metformin, a drug against several symptoms of the metabolic syndrome, as a potential repurposed drug against hearing loss (Milon et al., 2021). In research fields other than auditory, single cell deep sequencing and analytical tools such as RNA velocity, BRIE2, Cell2Cell communication tools (e.g., Cellphone DB; CellChat), and bifurcation analysis (e.g., scVelo and scFates) have allowed an unprecedented understanding of neural development and neurologic disease progression, which is of high value for the discovery of highly specific and effective drugs (Faure et al., 2022). As a consequence, benefits for the pharmacological R&D pipeline in the auditory field may soon emerge from the growing knowledge that has been acquired in the last decade.

### A. Mapping the Cochlear Cellular Landscape with Single-Cell RNA Sequencing

Historically, the complex architecture of the cochlea, given its multiple compartments and dense bony structure, and the low survival rate of murine hair cells after sorting have made it challenging to obtain a comprehensive cellular picture of the cochlea at different stages of its maturation. Table 1 presents, to the best of our knowledge, a comprehensive picture of the published single-cell RNA sequencing scRNAseq studies in rodents. It evidences the wide range of developmental stages, cochlear compartments collected, and strains and backgrounds but also sequencing methods used, the latter of which are key in revealing not only top cellular markers but also more complex mechanisms in cellular function. For instance, the four neuronal subtypes described in Section II and identified by two different laboratories, which used an advanced and full-read RNA sequencing platform called Smart-seq2 on fewer than 500 cells (Petitpré et al., 2018), have never been captured in the commonly used 10X Genomics, even when more than 5000 sorted cells were used (Rai et al., 2020; Sanders and Kelley, 2022; Jean et al., 2023). Out of 24 studies, only 2 used the CBA/CaJ (Milon et al., 2021; Shrestha et al., 2018); the majority of the other studies have used CD-1 or C57BL/6 J mice, both prone to ARHL, which might explain why these focused mainly on the developmental aspects of the cochlea (Table 1). Possibly, one of the most comprehensive analyses ever performed on the adult cochlea is the study of Jean et al., who analyzed 88,006 cells at various stages of postnatal and adult development in C57BL/6 J mice (Jean et al., 2023). However, as commented earlier, not all SGN subtypes were captured, and batch-to-batch effects occurred as well (Jean et al., 2023). New technologies such as the Smart-seq3xpress (Hagemann-Jensen et al., 2022) may provide novel insights into the cellular biology within the cochlea that go beyond the numerous cell types already identified (Jean et al., 2023). These technologies may even provide some strong translational value on organoids and human fetal cochlear tissue that have recently been brought to single-cell technologies (Van Der Valk et al., 2023).

**TABLE 1:** Single-cell RNA sequencing studies on the cochlea Studies are listed in chronological order, displaying the cochlear tissue samples, the species and strain, the age at sample collection, the total number of cells used (c = cells; *n* = nuclei), and the sequencing method used.

Author	Year	Tissue	Species (Strain)	Age	# Tot Cells	Method	Ref
Burns et al.	2015	Cochlear epithelium	Mice (CD1)	Neonatal	97c		Burns et al., 2015
Petitpré et al.	2018	SGN	Mice (C57BL/6 J)	P3	478c	Smart-seq2	Petitpré et al., 2018
Shrest5/11/24 9:54:00AMha et al.	2018	SGN	Mice (C57BL/6 J; CBA/CaJ)	P25-27	186c	Smart-seq2	Shrestha et al., 2018
Ranum et al.	2019	isolated IHC, OHC, DC	Mice (C3HeB/FeJ)	P15	132c	Smart-seq2	Ranum et al., 2019
Gu et al.	2020	Stria	Mice (CBA/J)	P30	5,681n	10 × Genomics	Gu et al., 2020
Kalra et al.	2020	Cochlea	Mice (CD1)	P2	3,411c	10 × Genomics	Kalra et al., 2020
Kolla et al.	2020	Cochlear duct	Mice (CD1)	E14, E16, P1, P7	30,670c	10 × Genomics	Kolla et al., 2020
Li et al.	2020	SGN, HC, glial	Mice (mutant ns)	P1, P8, P14, P30	?	Ovation RNA-Seq V2	Li et al., 2020
Rai et al.	2020	Cochlea	Mice (mutants*)	P12, P26, P33	5,470c	10 × Genomics	Rai et al., 2020
Chen et al.	2021	Cochlear duct	Rats (Sprague–Dawley)	P1, P7	28,557c	10 × Genomics	Chen et al., 2021
Milon et al.	2021	SGN, stria vascularis	Mice (CBA/CaJ; CBA/Ca/Sca)	2-4 m	69,117c	10 × Genomics	Milon et al., 2021
Wang et al.	2021	HC, SC	Mice (mutant ns)	P2	695c	10 × Genomics	Wang et al., 2021
Xue et al.	2021	Cochlea	Mice (C57)	P20	4,527c	10 × Genomics	Xue et al., 2021
Petitpré et al.	2022	SGN	Mice (mutant ns)	E14.5 - E18.5, P3	2,308c	Smart-seq2	Petitpré et al., 2022
Xu et al.	2022	Cochlea (upper half)	Mice (C57BL/6 J)	P14, P28	7,786c	10 × Genomics	Xu et al., 2022
Sanders et al.	2022	SGN	Mice (CD1)	E14, E16, E18, P1	5,441c	10 × Genomics	Sanders and Kelley, 2022
Iyer et al.	2022	Cochlea	Mice (mutant ns)	P8, P15	9,693c	10 × Genomics	Iyer et al., 2022
Piekna-Przybylska et al.	2023	SC	Mice (CBA/CaJxC57BL/6 J)	P2	∼300c	Smart-Seq Single +	Piekna-Przybylska et al., 2022
E. C. Boussaty et al., preprint, DOI: 10.1101/2023.02.15.528661	2023	Cochlea	Mice (CFW)	2, 6, 10 m	36,000n	10 × Genomics	Boussaty et al., 2023
Jean et al.	2023	Cochlea	Mice (C57BL6/J)	P8, P12, P20	88,006c	10 × Genomics	Jean et al., 2023
Jean et al.	2023	Cochlea	Mice (C57BL6/J)	P8, P12, P20	28,822n	10 × Genomics	Jean et al., 2023
Sun et al.	2023	Cochlea	Mice (C57BL/6 J)	1, 2, 5, 12, 15 m	45,972c	10 × Genomics	Sun et al., 2022
Koh et al.	2023	Stria vascularis	Mice (Slc26a4+/+)	P22, P42	138c	Smart-seq2	Koh et al., 2023
Liu et al.	2023	Organ of Corti	Mice (C57BL/6 J)	P7	9845c	10 × Genomics	Liu et al., 2023

DC, deiter’s cells; IHC, inner hair cells; OHC, outer hair cells; SC, support cells; SGN, spiral ganglion neuron.

* mutant strain background = 129xFVBxC57BL/6J

### B. Identifying Cellular Targets in Humans: The Emerging Benefits of Population Genetics

Preclinical incentives have been mainly focused on the regeneration of hair cells. However, the relative contribution of each cochlear region (e.g., HC, SGN, SV) in humans remains debated. This is of utmost translational relevance for defining R&D strategies tailored to patients’ needs. Due to the very difficult access to fresh cochlear material in humans, research has been limited to histological assessments, complexified by the artifacts caused by the nonimmediate preservation of postmortem tissue. With more recent refinements in histological preservation, the role of SV degeneration on ARHL, established by Schuknecht in the 1970s (Schuknecht et al., 1974), was challenged by a study from Wu et al. showing of 120 human cases a severe loss of hair cells at high frequencies with increasing age but also unexpectedly at low frequencies (Wu et al., 2020). A follow-up study from Kaur et al. using a larger number of human cases (*n* = 160) including more “flat-audiograms” confirmed the hypothesis that a flatter audiometric shape with a high degree of low-frequency hearing loss correlates with greater strial atrophy whereas a down-sloping “sensory” pattern correlated more with hair cell loss (Kaur et al., 2023). As not all forms of hearing loss are alike, predictions of the cellular source of hearing loss based on the audiogram are essential for improving the efficacy of future pharmacological interventions.

**Fig. 4:**
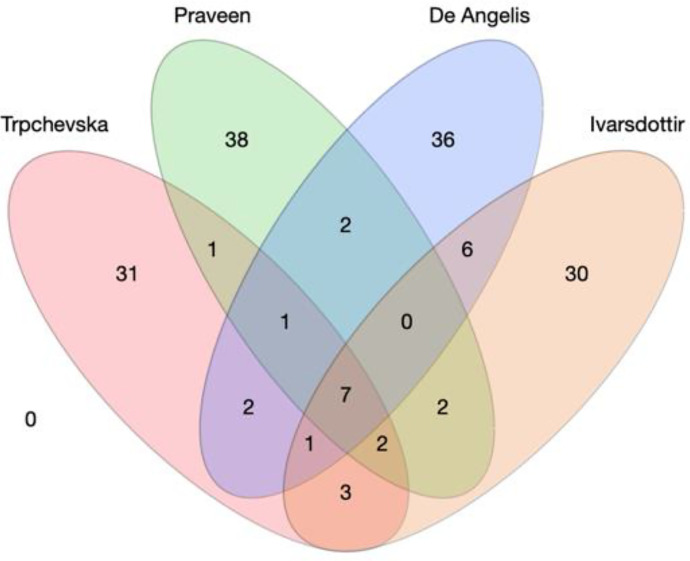
Current large genome-wide association studies on age-related hearing loss. (A) Four-dimensional Wenn Diagram with the number of overlapping loci reported in Trpchevska et al., 2022; Praveen et al., 2022; De Angelis et al., 2023; and Ivarsdottir et al., 2021. Seven loci appear common to the four studies (rs10901863, rs11238325, rs143282422, rs36062310, rs6545432, rs67307131, rs9493627).

Defining the contribution of a cochlear region based on histology has its own limitation: the functional changes of the region in question may precede the histological evidence. To provide molecular evidence of the cellular origins of ARHL, recent research combined large genome-wide association studies (GWAS) in humans (Table 2) with single-cell cochlear transcriptomics derived from some of the animal work described earlier. The assumption is that the most relevant cells for a given trait would robustly express the disease risk genes, as has been demonstrated in schizophrenia (Skene et al., 2018). Thanks to the availability of UK Biobank, pioneering work by Kalra et al. suggested a primary role for cochlear epithelial cells in human ARHL using scRNAseq data from postnatal day 2 CD-1 mouse cochlear ducts (Kalra et al., 2020). However, this study combined four definitions of hearing abnormality (i.e., hearing difficulty, the use of hearing aids, speech in noise difficulties, and tinnitus) in the GWAS analysis, and the scRNAseq analysis included the SV. A follow-up study from Trpchevska et al. (2022) increased the sample size to 723,266 individuals and used the largest 10X-Genomics scRNAseq of adult mouse SGN and SV available at the time (>50,000 cells) (Milon et al., 2021), complementing it with another dataset using of mature mouse cochlea sample (∼ 100 cells from P15 cochleae) (Ranum et al., 2019). The analysis revealed an enrichment in spindle and root cells, suggesting a role for the SV in ARHL. With cochlear epithelial cells on the one hand and cells from the SV on the other, a recent study from Eshel et al. leveraged new GWAS data (Ivarsdottir et al., 2021; Praveen et al., 2022; De Angelis et al., 2023) and performed enrichment analyses using other scRNAseq datasets to propose that cells from the sensory epithelium, rather than the SV are the cell types involved in ARHL (Eshel et al., 2024). Although Eshel et al.’s study does not systematically compare all scRNAseq datasets with all available highly powered GWAS, it does suggest overall that the results from the enrichment analysis strongly depend on 1) the data source for the GWAS; 2) the enrichment method used [e.g., LDSC (Finucane et al., 2015); MAGMA (de Leeuw et al., 2015); scDRS (Zhang et al., 2022)]; 3) the scRNAseq technology used; and 4) the age of the sample. Indeed, among the genome-wide significant loci that were identified across the four GWAS, only seven were found common (Fig. 4), also indicating that the associations with ARHL may depend on 1) the studied populations, 2) the statistical software used, 3) the statistical method used to adjust for individual cohorts, and 4) the stringency of the meta-analysis (Table 2).

**TABLE 2:** GWAS on hearing lossStudies are listed in chronological order, displaying the trait, sample size, cohorts used, number of loci identified, and software used.

Author	Year	Trait	*n* (Case/Controls)	Source	# Loci	Software for GWAS	Reference
Hoffmann et al.	2016	ARHI	6,527/45,882	GERA	1	PLINK (logistic regression)	Hoffmann et al., 2016
Wells et al.	2019	Hearing difficulty	87,056/163,333	UKBB	41	BOLT-LMM (LMM)	Wells et al., 2019
Wells et al.	2019	Hearing aid	13,178/240,740	UKBB	7	BOLT-LMM (LMM)	Wells et al., 2019
Nagtegaal et al.	2019	Audiometry	9,675/356,141	CHARGE consortium (6)	7	ProbABE, PLINK, GeneABEL, GENESIS, EMMAX (LMM and linear regression)	Nagtegaal et al., 2019
Kalra et al.	2020	Hearing difficulty, hearing aid, speech-in-noise, tinnitus	x/330,759	UKBB	31	Hail (linear regression)	Kalra et al., 2020
Liu et al.	2021	Hearing difficulty, hearing aid, speech-in-noise, tinnitus	x/362,396	UKBB	35/22/2/11	PLINK (logistic regression)	Liu et al., 2021
Ivarsdottir et al.	2021	ARHI	121,934/591,699	Iceland/UKBB	45	Not-reported (logistic regression)	Ivarsdottir et al., 2021
Trpchevska et al.	2022	ARHI self-report and ICD10	147,997/575,269	UKBB, AGES, DTR, EGCUT, FinnGen, FHS, HABC, INGI-FVG, RS, SA, SALT(Y), STAGE, TwinsUK, WGHS	48	PLINK, MLMA, SAIGE, GEMMA, Rvtests, Mach2Dat, BOLT-LMM (linear regression, LMM)	Trpchevska et al., 2022
Praveen et al.	2022	Hearing problems (hearing difficulty, hearing aid, speech-in-noise) and ICD10	125,749/469,497	UKBB, FinnGen, MALMO, SINAI, GHS	53	Regenie (LMM)	Praveen et al., 2022
De Angelis et al.	2023	Hearing problems (Hearing difficulty, hearing aid, speech-in-noise)	145,529/356,296	UKBB, NHS I, NHS II, HPFS, MVP	54	PLINK (logistic regression)	De Angelis et al., 2023

ARHI, age-related hearing impairment; GWAS, genome-wide association studies; ICD10, International Classification of Diseases, Tenth Revision.

These studies highlight the potential of merging human genetics with mouse scRNAseq to better understand the cellular origins of ARHL in humans. However, the debate remains open. We believe that the next logical step would be a coordinated effort to expand the sample size with an optimized phenotypic definition of ARHL and a statistically comprehensive and harmonized GWAS meta-analysis of all cohorts. The improved methods for the isolation and sorting of cells from the whole adult mouse cochlea using deep-sequencing technologies are encouraging and could increase the power of the statistical analyses. The implementation of such tools to human cochlear material will be a necessary step to determine the cellular targets for ARHL.

## VII. Future Considerations in Preclinical Research Designs to Enhance Translational Validity

A number of clinical failures have occurred in some of the earliest incentives against hearing loss, including AUT00063, AM-101, SENS-401, OTO-311, and FX-322. Can any lessons be learned from other fields to increase the likelihood of success in the auditory field? A major analysis for the attrition of drug candidates from four big pharmaceutical companies (AstraZeneca, Eli Lilly, GlaxoSmithKline, and Pfizer) (Waring et al., 2015) led to the establishment of the five-dimensional framework from AstraZeneca (Morgan et al., 2018). Briefly, the key features for successful drug development are 1) the right target, 2) the right tissue, 3) the right safety, 4) the right patient, and 5) the right commercial potential. This is where programs containing uncertainty at one of these levels were ended to minimize the risk of failures in larger and more costly clinical trials. Consequently, of the 287 programs aimed at the discovery of small molecules, only 76 were continued. In 10 years, the field has witnessed a fantastic rise in clinical programs for hearing loss, with 8 programs reported in 2013 (Sheridan, 2013) versus 60 programs solely on pharmacology in early 2024 (Table 3). The recent successful restoration of hearing using gene therapy in children with mutations on the *OTOF* gene has set the bar high (Lv et al., 2024) and illustrates what can be achieved, at least for some specific, well-defined monogenic disorders. As most hearing pathologies will not benefit from such homogenous and monogenic phenotype, more fundamental, translational and applied research will be needed for optimizing treatment of the global population.

**TABLE 3:** Active clinical development programs for the pharmacological treatment of hearing loss Current development programs of drug candidates for the treatment of hearing loss (where “in development” is defined as, at minimum, a disclosed, identified target in active development) were identified in the drugs database of global data (January 2024 cutoff) and cross-referenced with company websites. Gene therapy is excluded.

Company	Hearing Loss Indication	Brand or Drug Name (Active Principle)	Highest Active Development Stage
Fennec Pharmaceuticals Inc.	Cisplatin-induced ototoxicity (pediatric)	Pedmarky (sodium thiosulfate)	Marketed
Sound Pharmaceuticals Inc.	Ménière’s disease/aminoglycoside-induced ototoxicity	SPI-1005/SPI-3005 (ebselen)	Phase III/II
Acousia Therapeutics GmbH	Cisplatin-induced ototoxicity	ACOU085	Phase II
AudioCure Pharma GmbH	Idiopathic sudden SNHL	AC-102	Phase II
Gateway Biotechnology Inc.	NIHL	GW-HP1 (zonisamide)	Phase II
Nobelpharma Co Ltd	Pendred syndrome/DFNB4 (hearing loss)	NPC-12 (sirolimus)	Phase II
Sensorion SA	Sudden SNHL/cisplatin-induced ototoxicity/hearing preservation after cochlear implantation	SENS-401 (arazasetron)	Phase II
Spiral Therapeutics Inc.	Ménière’s disease	SPT-2101 (dexamethasone)	Phase I
Audion Therapeutics BV	SNHL	AUD-1001 (LY-3056480)	Phase I
IMD Farm Co Ltd	SNHL	IMDSST-03	Phase I
Otologic Pharmaceuticals Inc.	SNHL	NHPN-1010 (acetylcysteine + disufenton sodium)	Phase I
Regeneron Pharmaceuticals Inc.	Cisplatin-induced ototoxicity	DB-020 (sodium thiosulfate)	Phase I

NIH, noise-induced hearing loss; SNHL, sensorineural hearing loss.

In this last section, we emphasize the sixth R, “the right time” of treatment—something that is known as chronopharmacology, which should become an element in preclinical research. In this regard, several circadian researchers joined efforts to emphasize the need to control for the timing of drug delivery to evidence the benefits and minimize the side effects (Cederroth et al., 2019a). While treatment at different times of the day has been explored in humans, it has less commonly been explored in preclinical work. This could be a reason for the low correspondence between preclinical data and clinical trials: rodents are nocturnal, and humans are diurnal. An increased awareness about circadian biology is needed for the translation of preclinical data.

### A. The Circadian System: Entering the Fourth Dimension

The circadian system has been highly conserved throughout evolution and is a process that dictates phases of activity and rest across the entire body, most often in response to the daily cycles of light and darkness. In mammals it is organized hierarchically, with the bilateral suprachiasmatic nuclei (SCN) of the hypothalamus being the master clock regulating nearly all bodily functions (Kalsbeek et al., 2006; Mohawk et al., 2012). The SCN, the master clock, orchestrates the rhythmicity of peripheral organ function through both sympathetic and parasympathetic pathways and, when the SCN is ablated, peripheral tissues become asynchronous (Yoo et al., 2004). Light is the main entrainment cue for SCN circadian rhythms, but other cues like feeding, locomotor activity, temperature, and hormonal factors can also synchronize peripheral organs. Molecularly, cells contain an autoregulatory transcriptional/translational feedback loop, namely the core clock genes *Per, Cry, Clock, Bmal1, Rev-erβ,* and *Ror* (Bass and Takahashi, 2010). The tight coordination of the positive and negative elements of transcription, as well as post-transcriptional and post-translational modifications, impose time delays that produce an accurate and robust cellular oscillator with a 24-h periodicity (Reppert and Weaver, 2002). A disruption of clock genes (*Per, Cry, Clock, Bmal1, Rev-erβ,* and *Ror*) in mice is known to generate a variety of phenotypes (Ko and Takahashi, 2006). Disruption of *Bmal1* has the greatest impact on clock rhythms and triggers a wide array of disorders including arrhythmic locomotor activity in constant darkness, arthropathy (Bunger et al., 2005), infertility (Alvarez et al., 2008), symptoms of the metabolic syndrome (Lamia et al., 2008), reduced B-cell production (Sun et al., 2006), and decreased lifespan (Kondratov et al., 2006), overall highlighting its important role in maintaining homeostasis. Thus, it is no surprise that some diseases peak in their symptom severity at different times of the day (Table 4).

**TABLE 4:** Timing of symptom severity for a selected set of diseases There are several diseases and disorders that exhibit symptoms based on the time of day.

Disease	Reference
Morning	
Rheumatoid arthritis	Gibbs and Ray, 2013
Heart attacks and myocardial infarction	Muller, 1999
Migraine headaches	Fox and Davis, 1998
Allergy	Gelfand, 2004
Infection	Long et al., 2016
Evening	
Peptic ulcers	Jamali et al., 1995
Hypercholesterolemia	Saito et al., 1991
Temporomandibular joint pain	van Grootel et al., 2005
Asthma	Bohadana et al., 2002
Epilepsy	Pavlova et al., 2004

#### 1. The Time of the Day When Glucocorticoids and Circadian Rhythms Meet

Glucocorticoids (GCs) are steroid hormones that are secreted from the adrenal glands and regulated by the stress-responsive hypothalamic-pituitary-adrenal axis (Chrousos, 1995). GCs play an important role in regulating glucose formation in the liver, maintaining homeostasis, and regulating the immune system and stress-related physiology (Munck et al., 1984). They are released in a circadian manner, with a peak concentration found just prior to the onset of the active phase in humans and rodents (daytime for humans and nighttime for rodents when the activity is high) and reaching a minimum during the inactive state. When the SCN is ablated, the secretion of the hormone becomes arrhythmic, demonstrating that GC secretion is under circadian control (Moore and Eichler, 1972; Stephan and Zucker, 1972; Ishida et al., 2005; Radziuk, 2013). GCs, in turn, also entrain the rhythmicity of peripheral organs (Mohawk et al., 2012; Challet, 2015). Indeed, exposing fibroblasts to dexamethasone results in a robust circadian induction of *Per1* gene expression, and a phase shift is observed when the drug is applied during the descending phase of corticosterone secretion (Balsalobre et al., 2000). Conversely, clock genes also regulate glucocorticoid homeostasis. Mice with either *Bmal1* or *Clock* mutations show hypercortisolism at the onset of darkness (Turek et al., 2005; Leliavski et al., 2014), and *Per2* mutant mice display arrhythmic glucocorticoid release (Zhang et al., 2011). Overall, a close bidirectional interaction occurs between the clock and the glucocorticoid systems.

### B. Glucocorticoid Receptors in the Cochlea

Glucocorticoids exert their diverse effects through a specific intracellular receptor, the glucocorticoid receptor (GR), which belongs to the nuclear receptor superfamily and is ubiquitously expressed in almost all human tissues and organs. Unliganded GR is sequestered in the cytoplasm within a complex of chaperones, and glucocorticoid binding induces a conformational change within the receptor, allowing its release from the chaperone complex, dimerization, and translocation to the nucleus where it modulates the expression of target genes. Once in the nucleus, the GR homodimers bind to the glucocorticoid response elements to regulate gene transcription. Therapeutic doses of glucocorticoids are successfully used for treating inflammatory and autoimmune diseases (Boumpas et al., 1993), and the influence of glucocorticoids on auditory function was first reported in the late 1970s, when a substantial improvement of hearing was obtained after therapy with two synthetic analogs of the glucocorticoid hormone (cyclophosphamide and dexamethasone) in patients with autoimmune hearing loss. GRs are widely distributed in the CNS and other organs (Herman et al., 2003; Androutsellis-Theotokis et al., 2013), but they have also been detected in the inner ear of animals and humans in both the cochlear and vestibular systems (Furuta et al., 1994). In the cochlea, GR is found in the hair cells, supporting cells, spiral ligament, and SV, indicating a possible role in the regulation of both sensory and nonsensory tissues within the cochlea. Cochlear GR expression is modulated in response to acoustic trauma or restraint stress (Rarey et al., 1995; Tahera et al., 2006), and glucocorticoid analogs (i.e., dexamethasone) protect against acoustic trauma, triggering a GR-dependent activation of NF*κ*B and MAPK signaling pathways (Tahera et al., 2006).

### C. Cochlear Clock Rhythms and Their Modulation by Dexamethasone

Since GCs are potent synchronizers of peripheral clocks (Dickmeis, 2009; Cuesta et al., 2015) due to their interactions with core clock proteins (Balsalobre et al., 2000; Lamia et al., 2011), it has been hypothesized that the cochlea would also display features of the circadian system. Pioneering work from Meltser et al. using the Nanostring technology (Vikhe Patil et al., 2015) showed that the cochlea expresses elements of the autoregulatory transcriptional/translational feedback loop, namely the core clock genes *Per1, Per2, Bmal1*, and *Rev-erbα* (Meltser et al., 2014). PER2 is abundantly expressed in hair cells and SGNs, with a tonotopic gradient of circadian activity starting at the apical region and progressing toward the middle turn (Park et al., 2017). Using Period2-Luciferase (PER2::LUC reporter mice, Meltser et al. showed that cochlear explants display robust circadian oscillations that persist over 6 days ex vivo (Meltser et al., 2014). The cochlear clock is also under the control of the SCN, since bilateral SCN electrolytic lesions abolish rhythmicity in the cochlea (Cederroth et al., 2019). Dexamethasone (DEX), a synthetic GR agonist, increases the amplitude of PER2::LUC oscillations ex vivo when administered at trough of PER2 expression and has an opposite effect when given at the peak of the oscillations (Cederroth et al., 2019b). GR antagonists (RU486), but not mineralocorticoid receptor antagonists (spironolactone), blocked the effects of DEX. These findings indicate that the cochlea possesses a circadian system that is responsive to the modulation of GR activity.

### D. The Time of the Day Determines the Degree of Trauma in the Cochlea

Delivering the same noise trauma to CBA/Ca/Sca mice during the inactive (daytime) or active (nighttime) phase results in different outcomes. Exposure to a noise trauma during the inactive phase causes a temporary threshold shift that fully recovers after 2 weeks, whereas an exposure during the active phase causes a permanent threshold shift (Meltser et al., 2014). This circadian vulnerability to auditory damage is also seen with aminoglycosides (Yonovitz and Fisch, 1991) and cisplatin (Tserga et al., 2020), where in the latter administration during the active phase leads to greater afferent synapse loss. Loss of the glutamate aspartate transporter function, which causes cochlear synaptopathy (Tserga et al., 2021), exacerbates the vulnerability to cisplatin administered during the active phase (Tserga et al., 2020). The circadian sensitivity to cochlear insults may, however, be dependent on the level of noise (or ototoxic drug) and also on the species, strain, and even substrain (Versteegh et al., 2022). Removal of the adrenal glands abolishes this differential day/night noise sensitivity (Cederroth et al., 2019), strongly supporting the notion that the increased vulnerability to noise seen during the active phase is related to the glucocorticoid system.

Concerning ototoxicity, it is rather surprising to see the low correspondence between preclinical data and the clinical trials. Most studies using mice show small changes in ABR threshold shifts (∼10 dB) after a single cisplatin injection (Kim et al., 2009), which leaves a small dynamic range for assessing the protective effects of drugs. Indeed, studies have demonstrated a greater vulnerability in rats and guinea pigs to aminoglycosides and cisplatin than in mice (Poirrier et al., 2010), which may explain, at least in part, why these larger rodents have been the preferred model in hearing loss preclinical development. However, a clear limitation to a single-bolus paradigm is that it does not mimic the multiple insults that humans receive in serial administrations. In this regard, the Cunningham laboratory developed a highly effective multicycle model of a single cisplatin administration (3.5 mg/kg) in CBA/CaJ mice, three times interspaced with a 1-week recovery period, leading to between 40 and 60 dB ABR threshold shifts at all frequencies, and a near complete loss of distortion products of otoacoustic emissions that persisted 4 months after treatment (Fernandez et al., 2019). Eventually, this model could become a preferred choice for R&D in the pharmaceutical industry, with prior validation in rats or guinea pigs, since mice are amenable to genetic manipulations and enable the validation of drug candidates by inactivating the target protein. It is still necessary, however, to incorporate a multicycle administration at nighttime, during the active phase, to optimize the translational value of this model and more closely mimic the exposure situation occurring in humans.

### E. The Time of the Day and the Effectiveness of Specific Drugs on the Cochlea

There is emerging evidence showing that some drugs are more effective at nighttime than at daytime, whereas the opposite is true for other drugs (Cederroth et al., 2019). An example of how the timing of treatment can improve the overall treatment outcomes in humans is provided by oxaliplatin. The first demonstration of its clinical efficacy in colorectal cancer was provided in a large phase II clinical trial using chronomodulated delivery (Lévi et al., 1992), a finding that was later confirmed in a randomized phase III trial (Lévi et al., 1997).

In the cochlea, the dependency of the differential sensitivity to day/night noise trauma and the different responses of the cochlear clock to DEX depending on day or night treatments ex vivo led to the hypothesis that DEX could also have differing outcomes with regards to the vulnerability to noise trauma depending on when the drug is administered. DEX was protective against acute noise trauma only when administered during the inactive phase, when circulating glucocorticoids are low, but not when administered during the active phase (Cederroth et al., 2019a). Conversely, the improved recovery from the noise exposure during the inactive phase was associated with the ability of the cochlea to increase *Bdnf* expression in response to noise, a phenomenon that did not occur during the active phase. Treatment with di-hydroxyflavone, an agonist of the BDNF receptor TrkB, effectively protected mice from noise trauma when delivered during the active phase but not during the inactive phase (Meltser et al., 2014). Had the compound not been tested at nighttime, the efficacy of this molecule would have been missed, and the fundamental relevance of circadian mechanisms and TrkB signaling in the treatment of hearing disorders would have been ignored. Table 5 provides some examples of cochlear circadian genes, which are the targets of drugs used in the auditory field. This concept, referred to as chronopharmacology, considers the dependencies between the time of drug administration and the overall biological effect, including side effects. Since identifying the appropriate time for drug administration is intimately related to endogenous circadian rhythms, the presence of a circadian clock in the cochlea could have important implications for drug discovery.

**TABLE 5:** Examples of genes that are circadian in the cochlea and are targets of drugs used in the auditory system “Reference” refers to the drugs acting on the target gene.

Drug	Target Gene	Function	Reference
Cisplatin	*Xpc*	Nucleotide excision repair	Kang et al., 2010
Gentamicin	*Lrp2*	Scavenger receptor, belonging to the low-density lipoprotein receptor family	Dagil et al., 2013; Kim and Ricci, 2022
Salicylate	*Cox-2*	Inducible enzyme (e.g., at sites of inflammation and cancer)	Turini and DuBois, 2002; Chen et al., 2015
Dexamethasone	*Nos2*	Induced by cytokines and lipopolysaccharide	Tunçtan et al., 2002
7,8-dihydroxyflavone	*Ho-1*	Member of the heat shock protein family and is stressed-induced	Henrich et al., 2023

A recent report by Plontke et al. reveals in a large three-arm, parallel-group, randomized, triple-blind clinical trial that even a high dose glucocorticoids does not show benefits against idiopathic sudden sensorineural hearing loss when compared with a low dose (Plontke et al., 2024). But is timing critical for drugs that have long half-lives in humans like dexamethasone? Some researchers argue yes (Zhang et al., 2014), but while we believe that this could apply for the majority of drugs, specific drugs may escape the rule. In our hands, DEX was shown to be more beneficial in protecting mice against noise trauma after administration during the inactive phase. Likewise, cisplatin—which is known to persist in the cochlea over months after a single injection—was revealed to be more harmful to the auditory synapse when administered during the active phase in mice (Tserga et al., 2020). Thus, even for dexamethasone, the quest for an optimal therapeutic intervention may not be over until timing is considered. Auditory researchers should take advantage of the favorable winds of change to include a crucial dimension in preclinical and clinical research: timing of the insult, timing of the drug intervention, and timing of the outcome measure.

### F. A New Science with In Vitro and In Vivo Models

In vitro and in vivo models may provide new resources for improving our understanding of the translational relevance of specific drugs targeting the auditory system. PER2::LUC mice are knock-in mice in which a *Luc* gene is fused in-frame to the 3′ end of the endogenous *mPer2* gene. Any organ from these mice can be collected and cultured ex vivo for the real-time monitoring of PER2 oscillations. This system has been used on cochlear explants to show the differential effects of di-hydroxyflavone or DEX treatment when applied during peak or trough PER2 expression (Meltser et al., 2014; Cederroth et al., 2019b), as well as to show the reversible blockade of cochlear rhythms by TEA (a K+ channel antagonist) and 1,2-Bis(2-aminophenoxy)ethane-N,N,N0,N0-tetraacetic acid (an extracellular calcium chelator), providing additional insights in the mechanisms controlling cochlear clock rhythms. This system can thus be used on the cochlea as well as on other organs with more abundant tissue such as the liver, although extrapolations should be done with caution [see examples in Cederroth et al. (2019b)] and allow for the assessment of the direct impact of a drug on the clock system (amplitude, period, and phase).

While there are a large number of cellular in vitro systems, nearly equivalent to all organs of the body, the hearing field has suffered from a lack of such tools not only to facilitate fundamental research but also to allow validation in drug screens on cell systems closer to the target organ. Historically, the HEI-OC1 cells from the Kalinec group at the House Ear Institute have pioneered this research area by immortalizing cells from the OC using the *immortomouse* (Jat et al., 1991). These cells would proliferate at 33°C and 10% CO_2_ (the so-called permissive conditions), but when moved to 39°C and 5% CO_2_ (nonpermissive), they would differentiate into more advanced/mature cell types (Kalinec et al., 2003). However, due to the large amount of cell death occurring during the culture transition to nonpermissive conditions, the toxicity to aminoglycosides or cisplatin could not be demonstrated as easily. As a consequence, this cell line has mostly been used in a permissive condition, during more immature stages and high proliferation. While initially these cells showed high sensitivity to aminoglycosides when compared with NIH3T3 fibroblast cells (Kalinec et al., 2003), they progressively lost this sensitivity while maintaining the sensitivity to cisplatin (Cederroth, 2012). Nonetheless, HEI-OC1 cells have been used in several studies to identify the signaling pathways involved in cisplatin-mediated cell death. However, we found that ATP luminescence-based viability assays and Caspase-3 assays—the most commonly used tools for assessing cisplatin ototoxicity in HEI-OC1 cells—do not always reflect proper cell survival (Cederroth, unpublished observations). Indeed, protective drugs that are involved in mitochondrial function may boost ATP metabolism and lead to the false interpretation that viability has been improved. Likewise, a lower activation of Caspase-3 using protective drugs against cisplatin ototoxicity does not necessarily lead to lower apoptosis. Indeed, flow cytometry combined with ImageStream has revealed that Annexin V positive and Propidium Iodide positive cells (AnxV^+^/PI^+^) harbor not only apoptotic but also necrotic cells after cisplatin treatment, which may trigger confounding effects in the viability and Caspase-3 assays. Therefore, it is a combination of assessment tools that should be supportive of a protective mechanism in drug screening (Cederroth, 2012). The Kalinec laboratory published recommendations on how to culture these cells in optimal conditions, depending on the research questions (Kalinec et al., 2016).

Complementing the use of in vitro cell lines is use of the zebrafish (*Danio rerio*), which has become a replacement model for rodents, allowing for broad screens in a living system (Patton et al., 2021). Moreover, its potential use in the context of ototoxicity has gained attention in the past decades (Chiu et al., 2008). As mentioned, it was at the origin of the identification of PROTO-1 (Owens et al., 2008) and a deeper characterization of the improved chemical variant ORC-13661 showing protective effects (Kitcher et al., 2019). The benefits of the zebrafish as a model of cisplatin ototoxicity have been also demonstrated (Ou et al., 2007; Lee et al., 2022b; Thomas et al., 2013), with some initial drug screens performed (Wertman et al., 2020). However, to our knowledge, the findings related to cisplatin-mediated ototoxicity have not yet been pursued beyond these findings.

Exciting endeavors have recently arisen to develop organoids derived from induced pluripotent stem cells (iPSCs) from humans (Pianigiani and Roccio, 2024). Through a timed series of small molecule guided differentiation, it has been possible to generate three-dimensional cell aggregates (the so-called organoids) that have a remarkable resemblance to inner ear organs. This is a major breakthrough in the field since it enables researchers to bypass the inaccessibility of adult human cochlear tissue for primary cultures. Recent studies have performed in depth-characterizations using single-cell RNA sequencing technologies and compared these human iPSC-derived organoids with human inner ear embryonic tissue (Doda et al., 2023; Van Der Valk et al., 2023). A small limitation persists, however, in that the hair cells from the organoids have a closer identity to vestibular hair cells than to cochlear hair cells (Liu et al., 2016), and hair cell maturation can reach functionality after 150 to 200 days in culture, corresponding to week 18 to 20 of fetal inner ear development (Moore et al., 2023). This is consistent with the notion that iPSC-derived organoids in general maintain a fetal identity (Kim et al., 2020; Corsini and Knoblich, 2022), something that is less seldom seen when using primary cultures from either fetal or adult tissue (Park et al., 2022; Hendriks et al., 2023). As a consequence, while iPSC-derived inner ear organoids may not yet be suitable for high throughput screening (HTS), they are powerful tools for demonstrating target engagement in human tissue, assessing toxicity, or to validate the translational relevance of specific gene therapies (M. V. Ivanchenko et al., preprint, DOI: 10.1101/2023.11.09.566447). Recent advances in retinal organoids provide evidence of the large potential of inner ear organoids in drug R&D (Cowan et al., 2020; Spirig and Renner, 2024).

## VIII. Conclusions

The recent fundamental and translational advances in the auditory field are paving the way for some exciting years to come. These advances will not, however, come without hurdles. The difficulty of accessing the cochlear structure whether it be for collecting biopsies or samples or for the precise/quantitative drug delivery will remain a major technical challenge. Likewise, the many structures that the cochlea has and the many cells that comprise each of them illustrate the large heterogeneity in hearing loss phenotypes. The evidence collected in this review is a call for otolaryngology, head and neck surgery clinics to start biobanking temporal bones for histological and molecular analysis, collecting IPSCs for developing patient-derived organoids, obtaining DNA, and joining large consortiums to increase genetic knowledge at a global scale. The current biobanks do not have the required phenotypic resolution to address the existing knowledge gaps. There, hearing loss is often self-reported, and audiograms > 4 kHz are seldom available. We strongly believe that therapeutic success can only be achieved through large collaborations and converged efforts to method and statistical standardization, consensus, and data sharing. If gene therapy for hearing loss has the advantage of specifically targeting a cell type for a given monogenic disorder, pharmacological drugs have the benefits of having a broader action likely more suitable for more common forms of hearing loss such as those caused by aging, noise trauma, or ototoxicity. Their prevention has been a research quest for the past decades, but it is now time to focus on posttrauma therapy, as well as the regeneration of HCs and SGNs and the long-time ignored SV atrophy. The optimization of in vitro models will undoubtedly contribute to accelerating these advancements. Lastly, all preclinical work will have limited translational value unless it considers the time of the day for drug delivery, and the full potency of specific drugs may be underestimated if delivered at the wrong time of the day.

## Abbreviations

ABR: auditory brainstem response
AAV: adeno-associated virus
ARHL: age-related hearing loss
BBB: blood-brain barrier
BLB: blood-labyrinth barrier
JNK: c-Jun N-terminal kinase
CM: cisterna magna
CSF: cerebrospinal fluid
DEX: dexamethasone
EP: endocochlear potential
GWAS: genome wide association study
GC: glucocorticoid
GR: glucocorticoid receptor
IHC: inner hair cell
iPSC: induced-pluripotent stem cell
LOC: lateral olivocochlear
MET: mechanotransduction
NAC: *N*-acetylcysteine
NOX: NAPDH oxidase
OC: organ of Corti
OHC: outer hair cell
PER2::LUC: Period2 Luciferase
ROS: reactive oxygen species
RWM: round window membrane
SCN: suprachiasmatic nucleus
scRNAseq: single-cell RNA sequencing
SGN: spiral ganglion neuron
STS: sodium thiosulfate
SR: spontaneous rate
SV: stria vascularis
TRP: transient receptor potential.
